# Prevalence of latent tuberculosis infection and incidence of active tuberculosis in school close contacts in Shanghai, China: Baseline and follow-up results of a prospective cohort study

**DOI:** 10.3389/fcimb.2022.1000663

**Published:** 2022-09-23

**Authors:** Xiao Xiao, Jing Chen, Yue Jiang, Peng Li, Jin Li, Liping Lu, Yameng Zhao, Lihong Tang, Tianyuan Zhang, Zheyuan Wu, Lixin Rao, Zheng’an Yuan, Qichao Pan, Xin Shen

**Affiliations:** ^1^ Division of Tuberculosis and HIV/AIDS Prevention, Shanghai Municipal Center for Disease Control and Prevention, Shanghai, China; ^2^ Shanghai Institutes of Preventive Medicine, Shanghai, China; ^3^ Department of Tuberculosis Control, Shanghai Pudong New Area Center for Disease Control and Prevention, Shanghai, China; ^4^ Department of Tuberculosis Control, Songjiang District Center for Disease Control and Prevention, Shanghai, China; ^5^ Department of Tuberculosis Control, Minhang District Center for Disease Control and Prevention, Shanghai, China; ^6^ Shanghai Municipal Center for Disease Control and Prevention, Shanghai, China

**Keywords:** close contacts, latent tuberculosis infection (LTBI), prevalence, tuberculosis, incidence, QuantiFERON-TB Gold

## Abstract

**Background:**

The management of latent tuberculosis infection (LTBI) is a key action for the realization of the “End tuberculosis (TB) Strategy” worldwide, and it is important to identify priority populations. In this prospective cohort study, we evaluated the prevalence of LTBI and incidence of active TB among close contacts and explored the suitable TB control strategy in schools.

**Methods:**

We designed a cohort with 2 years of follow-up, recruiting freshman/sophomore TB patients’ close contacts from three administrative districts in Shanghai. These were chosen based on different levels of TB incidence reported in 2019. Questionnaires were included and all participants received both tuberculin skin test (TST) and QuantiFERON-TB Gold (QFT) at baseline, then tracked the outcomes of them during the follow-up period.

**Results:**

The prevalence of LTBI was 4.8% by QFT. Univariate analysis showed that the risk of LTBI was higher in those contacting bacteriologically confirmed patients or did not have BCG scars, including smokers. The risk increased with poor lighting and ventilation conditions at contact sites. Multivariate analysis showed that those contacting with bacteriologically confirmed patients (OR=4.180; 95%CI, 1.164-15.011) or who did not have BCG scars (OR=5.054; 95%CI, 2.278-11.214) had a higher risk of being LTBI, as did the current smokers (OR=3.916; 95%CI, 1.508-10.168) and those who had stopped smoking (OR=7.491; 95%CI, 2.222-25.249). During the 2-year follow-up period, three clinically diagnosed cases of TB were recorded, the 2-year cumulative incidence was 0.4% (95%CI 0.1-1.2), the median duration for TB occurrence was 1 year, the incidence rate of active TB was 2.0 per 1000 person-years with a total of 1497.3 observation person-years. For those LTBI, no one initiated preventive treatment, in the QFT (+) cohort, 1 TB case was observed, 71 person-years with an incidence rate of 14.1 14.1 (95%CI 2.5-75.6) per 1000 person-years, in the TST (+++) cohort, 2 TB cases were observed 91.5 person-years with an incidence rate of 21.9 (95%CI 6.0-76.3) per 1000 person-years.

**Conclusions:**

The results suggest that school close contacts are one of the key populations for LTBI management. Measures should be taken to further reduce the prevalence of LTBI and the incidence of active TB among them.

## Introduction

Tuberculosis (TB) is one of the leading causes of death from infectious diseases, World Health Organization (WHO) has recommended the “End TB Strategy” with a target to reduce the incidence of TB by 90% between 2015 and 2035 (World Health Organization, 2021). To realize this target, more effort is needed. Latent tuberculosis infection (LTBI) is a state of persistent immune response triggered by *Mycobacterium tuberculosis* antigens with no evidence of the clinical manifestation of active TB. A quarter of the world’s population is estimated to be LTBI. Better LTBI control is needed if the “End TB Strategy” goal is to be realized (World Health Organization, 2018).

WHO recommended key groups for LTBI management, emphasizing consideration of priority management groups in different countries (World Health Organization, 2018). In 2020, the Chinese Center for Disease Control and Prevention (China CDC) published a technical guide for tuberculosis control in China, which included close contacts of active TB patients in schools as one of the key groups for LTBI control (Liu et al., 2021). China has monitored school close contacts for years as keeping children healthy is important. However, there is limited evidence for the prevalence of LTBI and the outcomes of close contacts of active TB patients in schools in China.

Shanghai is located in eastern China. It is a high-income city and has a low TB incidence with a registration rate of TB cases of 26.7 per 100000 population, and 14.8 per 100000 students in 2019. Generally, approximately 300 student cases arise every year, and >60% of these cases are from colleges.

An emergency response mechanism was initiated by China CDC. When a student TB patient occurs, the public health practitioner responsible for the management of TB patients in the corresponding administrative district gets the case information in the fastest time through several approaches including a national TB registration system, and interprovincial notification of student TB cases, and self-reporting. Case investigation starts immediately and close contacts are identified according to the transmissibility of the index case and the intensity and duration of each contact’s exposure to the index case. Close contacts >15-years-old undergo a screening process consisting of inquiry of suspicious pulmonary tuberculosis (PTB) symptoms, tuberculin skin test (TST), and chest radiography examination. For contacts <15-years-old, chest radiography is only required when they have suspected symptoms or positive results of the TST test. Suspected symptoms referred to whether the individual had a cough, sputum ≥2 weeks, hemoptysis, bloody sputum, chest pain, trouble breathing, fever, night sweats, fatigue, or loss of weight and appetite.

Based on the results of screening, we divide close contacts into four categories: TB patients, presumable TB patients, individuals with LTBI, and medical observation subjects. Further diagnosis or treatment is required for presumable TB patients or TB patients. Preventive treatment is recommended for LTBI, and medical observation subjects are questioned about suspected symptoms at the 3,6, and 12 months after initial screening. For those refusing preventive treatment for LTBI, chest radiography is required at the 3,6, and 12 months after initial screening.

We launched a prospective cohort study in college close contacts in 2019 to elucidate the prevalence of LTBI including possible associated risk factors, and to track the development of active TB in Shanghai.

## Methods

### Sample size

The equation for calculating sample size was N=μ_α_^2^×pq/d^2^, where P was the prevalence of LTBI, q equaled 1-p, and d was the allowable error. As reported, the prevalence of LTBI among students between 17-18 years old was 6.2% by T-Spot (Hu et al., 2013), then made α=0.05, d=0.02. Considered a 10% non-response rate, got N=620.

### Study design and participants

According to different levels of incidence of TB reported in 2019, we chose three administrative districts (Minhang District, Pudong District, and Songjiang District) as study sites, where 36 (56.3%) of all 64 colleges in Shanghai are located in. We recruited freshman/sophomore close contacts of active TB patients in colleges and built a cohort with 2 years of follow-up to track outcomes.

All participants were >15-years-old. Routine screening methods included inquiry of suspicious symptoms of PTB, TST, and chest radiography. Participants filled in questionnaires and received a QuantiFERON-TB Gold (QFT) test, a commercial interferon-γ releasing assay (IGRA), after informed consent was obtained at baseline. The questionnaire information included age, sex, body mass index, BCG vaccination, smoking status, residence before entering college, lighting conditions, ventilation conditions, contact frequency/week, and contact duration. Every subject in the study received TST immediately after QFT. Based on the regular follow-up work for the contacts in 1 year, they were invited to receive chest radiography at the end of a 2-year follow-up.

### Definition

Bacteriologically confirmed TB case: Positive results of smear microscopy, culture, or GeneXpert MTB/RIF assay.

Clinically diagnosed TB case: Negative results of all the tests above, while had abnormal chest imaging and showed no response to anti-inflammatory treatment.

Active TB: Both bacteriologically confirmed cases and clinically diagnosed cases are defined as active TB.

Index case: The earliest case identified in an epidemic of PTB.

Close Contact: Person in close contact with the infectious index case during a likely period of infectiousness (**Table 1**).

**Table 1:** Ways for identifying close contacts.

Characteristics of the index case			Close contacts identification
Etiology positive	Cavitary chest radiograph	PTB symptoms	
Yes	Yes or No	Yes or No	3 months before the index case’s diagnosis, students who share the same class or dormitory with the index case, or stay with the index case in other possible confined spaces over 8 hours continuously or 40 hours cumulatively.
No	Yes	Yes or No
No	No	Yes
No	No	No	1 month before the index case’s diagnosis, students who share the same class or dormitory with the index case, or stay with the index case in other possible confined spaces for over 40 hours cumulatively.

M tuberculosis Infection tests and LTBI: TST was operated by trained nurses using the Mantoux method (Xiangrui; Beijing, China), intradermal injection of Tuberculin Purified Protein Derivative (TB-PPD; 0.1 ml, 5 IU). Induration at the TST site was read 72 hours later with a ruler or a caliper. QFT (Cellectis Ltd; Carnegie, Australia) was performed according to the manufacturer’s instructions with a cut-off value of 0.35 IU/mL or more. Those with TST induration ≥ 15 mm or positive results with the QFT test were recommended for preventive treatment. LTBI individuals were defined as those with positive QFT results after active TB was ruled out in this study.

Lighting condition: China is located in the middle and low latitudes of the northern hemisphere. The sun shines from the south all year round. We defined the lighting condition in the exposure site as poor, moderate, and good. Rooms with prolonged direct sunlight were defined as “good”, rooms with short direct sunlight were defined as “moderate”, and rooms without direct sunlight were defined as “poor”.

Ventilation condition: We defined the ventilation condition in exposure sites as poor and good. Rooms with cross-ventilated windows or with an indoor mechanical ventilation system were defined as “good”, otherwise, “poor” was considered.

### Statistical analysIs

Data were double entered in Epidata 3.1 software. Analysis was carried out by SPSS 20.0. Multivariable logistic regression models are constructed to realize the risk factors for LTBI. P<0.05 was considered statistically significant. Person-years were calculated from the time each subject was enrolled until a diagnosis of active TB, or at the end of the 2-year follow-up, accurate to years, and each month being 1/12 of a year.

## Results

### Study participation

In 2019, there were 19 TB epidemics in all study sites, resulting in 852 close contacts in total. Excluding 85 close contacts who rejected participating in the study, 767 eligible participants were included in the cohort, and data of all participants at baseline were available for analysis. During the 2-year follow-up period, 17 people were lost. Finally, we identified 750 follow-up subjects.

Among 19 index cases in the study, 12 were bacteriologically confirmed and 7 were clinically diagnosed. Of the 767 participants included in the cohort at baseline, 508 (66.2%) were in contact with an bacteriologically confirmed index case, the mean age was 19 ± 1 years old, 428 (55.8%) were male, 38.6% (n=296) of them were local students while 61.4% (n=471) lived in other provinces in China before entering college. 487 (63.5%) participants’ body mass index (BMI) were between 18.5 and 23.9, 130 (16.9%, BMI<18.5) were underweight and 105 (13.7%, BMI≥24) were overweight. 631 (82.5%) were nonsmokers and only 576 (75.1%) students had BCG scars (**Table 2**).

**Table 2:** Sociodemographic characteristics of subjects and exposure to the index cases in different districts.

	Total	Minhang District	Pudong District	Songjiang District
Age	19 ± 1	19 ± 2	19 ± 1	20 ± 1
Index case				
Clinically diagnosed	259 (33.8%)	50 (21.2%)	45 (15.3%)	164 (69.2%)
Bacteriologically confirmed	508 (66.2%)	186 (78.8%)	249 (84.7%)	73 (30.8%)
Sex				
Male	428 (55.8%)	121 (51.3%)	164 (55.8%)	143 (60.3%)
Female	339 (44.2%)	115 (48.7%)	130 (44.2%)	94 (39.7%)
BCG scar				
Yes	576 (75.1%)	203 (86.0%)	233 (79.3%)	140 (59.1%)
No	113 (14.7%)	32 (13.6%)	61 (20.7%)	20 (8.4%)
unexamined	78 (10.2%)	1 (0.4%)	0 (0.0%)	77 (32.5%)
BMI				
-18.4	130 (16.9%)	43 (18.2%)	51 (17.3%)	36 (15.2%)
18.5-23.9	487 (63.5%)	150 (63.6%)	181 (61.6%)	156 (65.8%)
24-27.9	105 (13.7%)	33 (14.0%)	40 (13.6%)	32 (13.5%)
28-	45 (5.9%)	10 (4.2%)	22 (7.5%)	13 (5.5%)
Residence before				
Local	296 (38.6%)	71 (30.1%)	129 (43.9%)	96 (40.5%)
Non-local	471 (61.4%)	165 (69.9%)	165 (56.1%)	141 (59.5%)
Smoking				
Non-smoker	631 (82.5%)	176 (74.9%)	252 (85.7%)	631 (82.5%)
Current smoker	96 (12.5%)	41 (17.4%)	36 (12.2%)	19 (8.1%)
Quit smoking	38 (5.0%)	18 (7.7%)	6 (2.0%)	14 (5.9%)
Lighting conditions				
Good	541 (71.3%)	214 (91.5%)	152 (51.9%)	175 (75.4%)
moderate	128 (16.9%)	18 (7.7%)	58 (19.8%)	52 (22.4%)
Poor	90 (11.9%)	2 (0.9%)	83 (28.3%)	5 (2.2%)
Ventilation conditions				
Good	563 (74.2%)	213 (91.0%)	172 (58.7%)	178 (76.7%)
Poor	196 (25.8%)	21(9.0%)	121 (41.3%)	54 (23.3%)
Contact frequency/week				
Less than 2 days	234 (30.8%)	55 (23.3%)	38 (12.9%)	141 (61.3%)
2-5 days	236 (31.1%)	110 (46.6%)	54 (18.4%)	72 (31.3%)
More than 5 days	290 (38.2%)	71 (30.1%)	202 (68.7%)	17 (7.4%)
Contact duration				
Less than 1 hour	358 (47.1%)	109 (46.2%)	66 (22.4%)	183 (79.6%)
1-2 hours	276 (36.3%)	71 (30.1%)	174 (59.2%)	31 (13.5%)
More than 2 hours	126 (16.6%)	56 (23.7%)	54 (18.4%)	16 (7.0%)

### Exposure to the index case

We visited all the exposure sites during the investigation such as the classroom, dormitory, or other confined spaces, and collected detailed data on exposure intensity and duration. Of all close contacts, 541 (71.3%) were exposed to good lighting conditions, and 563 (74.2%) were exposed to good ventilation conditions. The number of participants who reported “less than 2 days”, “2-5 days” and “more than 5 days” in terms of frequency contacting with index cases per week was 234 (30.8%), 236 (31.1%), and 290 (38.2%) respectively. The duration staying with the index case at a time was generally reported as “less than 1 hour” except for Pudong District (**Table 2**).

### Results of infection tests

**Figure 1:**
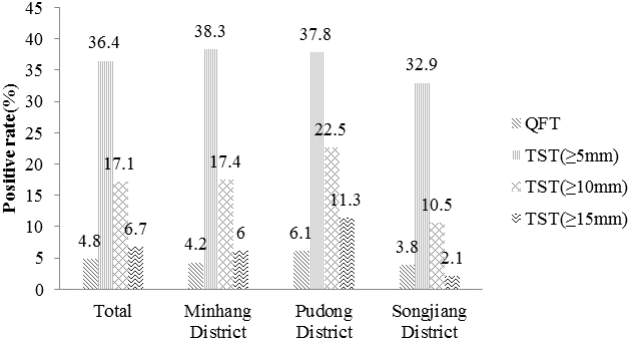
The positive rate of different *M. tuberculosis* infection tests. TST(≥5mm): an induration between 5 and 10 millimeters. TST(≥10mm): an induration between 10 and 15 millimeters. TST(≥15mm): an induration of 15 or more millimeters.

Overall, QFT was positive in 37 (4.8%) subjects. Excluding 20 subjects who did not undergo TST for the contraindications, the number of positive results was 272 (36.4%) when the cut-off value was 5 mm, 128 (17.1%) when the cut-off value was 10 mm, and 50 (6.7%) when the cut-off value was 15 mm respectively (**Figure 1**).

### Risk factors for LTBI

The prevalence of LTBI was 4.8% by QFT. Sociodemographic and exposure data were used to analyze the possible risk factors associated with LTBI. Univariate analysis showed that the risk of LTBI was higher in those contacting bacteriologically confirmed patients or did not have BCG scars, including the smokers. The risk increased with poor lighting and ventilation conditions at contact sites. By constructing multivariable logistic regression models, we found those in contact with bacteriologically confirmed patients (OR=4.180; 95%CI, 1.164-15.011) or did not have BCG scars (OR=5.054; 95%CI, 2.278-11.214) had a higher risk of LTBI, including the current smokers (OR=3.916; 95%CI, 1.508-10.168) and those who had quit smoking (OR=7.491; 95%CI, 2.222-25.249) (**Table 3**).

**Table 3:** Univariate and multivariate analysis of the risk factors associated with LTBI.

	Total	LTBI (%)	Crude OR^a^ (95%CI)	Adjusted OR^b^ (95%CI)
District		37 (4.8)		
Minhang District	236	10 (4.2)	1	1
Pudong District	294	18 (6.1)	1.474 (0.667-3.257)	1.232 (0.413-3.674)
Songjiang District	237	9 (3.8)	0.892 (0.356-2.237)	1.554 (0.455-5.305)
Index case				
Clinically diagnosed	259	6 (2.3)	1	1
Bacteriologically confirmed	508	31 (6.1)	**2.740 (1.128-6.656)** *	**4.180 (1.164-15.011)** *
Sex				
Male	428	18 (4.2)	1	1
Female	339	19 (5.6)	1.352 (0.698-2.619)	1.369 (0.589-3.183)
BCG scar				
Yes	576	18 (3.1)	1	1
No	113	16 (14.2)	**5.113 (2.521-10.371)** *	**5.054 (2.278-11.214)** *
unexamined	78	3 (3.8)	1.240 (0.357-4.310)	1.956 (0.348-10.993)
BMI				
-18.4	130	4 (3.1)	0.520 (0.179-1.511)	0.487 (0.160-1.480)
18.5-23.9	487	28 (5.7)	1	1
24-27.9	105	4 (3.8)	0.649 (0.223-1.892)	0.695 (0.227-2.129)
28-	45	1 (2.2)	0.373 (0.049-2.804)	0.340 (0.041-2.829)
Residence before				
Local	296	15 (5.1)	1	1
Non-local	471	22 (4.7)	0.918 (0.468-1.799)	0.765 (0.365-1.602)
Smoking				
Non-smoker	631	24 (3.8)	1	1
Current smoker	96	8 (8.3)	**2.299 (1.002-5.277)** *	**3.916 (1.508-10.168)** *
Quit smoking	38	5 (13.2)	**3.832 (1.375-10.683)** *	**7.491 (2.222-25.249)** *
Lighting conditions				
Good	541	22 (4.1)	1	1
moderate	128	5 (3.9)	0.959 (0.356-2.583)	1.025 (0.245-4.292)
Poor	90	10 (11.1)	**2.949 (1.374-6.457)** *	1.290 (0.226-7.368)
Ventilation conditions				
Good	563	22 (3.9)	1	1
Poor	196	15 (7.7)	**2.038 (1.035-4.013)** *	1.877 (0.453-7.786)
Contact frequency/week				
Less than 2 days	234	13 (5.6)	1	1
2-5 days	236	6 (2.5)	0.443 (0.166-1.187)	0.417 (0.129-1.346)
More than 5 days	290	18 (6.2)	1.125 (0.539-2.346)	0.670 (0.141-3.178)
Contact duration				
Less than 1 hour	358	16 (4.5)	1	1
1-2hours	276	16 (5.8)	1.315 (0.646-2.679)	0.708 (0.180-2.792)
More than 2hours	126	5 (4.0)	0.883 (0.317-2.463)	0.974 (0.206-4.595)

^a^OR, 95% CI, and P values were calculated in univariate logistic regression models.

^b^OR, 95% CI, and P values were calculated in multivariate logistic regression models.

*P < 0.05.

### The incidence of active TB in different groups

Among all of the 750 follow-up subjects, during the 2-year follow-up period, based on suspected symptoms screening and chest radiography examination, three clinically diagnosed cases of TB were recorded (**Table 4**). By the end of December 2021, the 2-year cumulative incidence was 0.4% (95%CI 0.1-1.2), the median duration for TB occurrence was 1 year, the mean annual incidence was 0.2%, the incidence rate of active TB was 2.0 per 1000 person-years with a total of 1497.3 observation person-years.

**Table 4:** Time characteristics of three incident TB cases.

NO.	Time of beginning observation	Time of being diagnosed	Time required for TB occurrence
PD0710	24 December 2019	24 September 2021	1.75 years
PD0741	24 December 2019	3 December 2020	1 year
SJ0111	26 April 2019	7 October 2019	Half a year

Details of the three clinically diagnosed cases of TB during the 2-year follow-up period were as follows, NO. PD0710 subject was a current smoker and came from a high TB burden area with the fifth-highest registration rate of TB in China, he had negative results in both TST and QFT tests at baseline, NO. PD0741 and NO. SJ0111 subjects were both overweight (BMI ≥24) local students, and had a TST induration ≥ 15 mm at baseline, NO. PD0741 subject had a positive result of QFT at baseline as well (**Table 5**).

**Table 5:** Sociodemographic characteristics of three incident TB cases.

	PD0710	PD0741	SJ0111
Relationship	Roommate	Classmate	Roommate
Sex	Male	Male	Female
Age	19	19	20
BMI	18	**24.8**	**24.7**
BCG scar	Yes	Yes	Yes
Smoking	**Current smoker**	Never smoker	Never smoker
Migrant	Yes	No	No
Address&registration rate of TB cases per 100000 population in 2019^◎^	**Hainan province&** **90.2/100000**	Shanghai & 26.7/100000	Shanghai& 26.7/100000
Baseline TST*	–	**+++**	**+++**
Baseline QFT	–	**+**	–

*TST(-): an induration of 5 or fewer millimeters. TST(+): an induration between 5 and 10 millimeters. TST(+++): an induration of 15 or more millimeters.

^◎^The registration rate of TB cases was 55.6 per 100000 population in China in 2019.

Of 750 follow-up participants, 36 were QFT (+) and 47 were TST (+++) at baseline, no one initiated preventive treatment, in the QFT (+) cohort, 1 TB case was observed 71 person-year with an incidence rate of 14.1 (95%CI 2.5-75.6) per 1000 person-years, in the TST (+++) cohort, 2 TB cases were observed 91.5 person-years with an incidence rate of 21.9 (95%CI 6.0-76.3) per 1000 person-years.

## Discussion

*M. tuberculosis* is transmitted by aerosol. Once exposed, a portion of the population can clear the bacterium by human innate and/or acquired immune responses (Drain et al., 2018), while, others may be infected. Approximately 5% of people infected will progress rapidly to active TB. The other 95% of people infected can develop a latent infection state and remain at risk for reactivation (Cadena et al., 2017).

Schools are crowded spaces. Long-term close contact in confined spaces provides conditions for the spread of *M. tuberculosis*. Once an infectious TB patient is present, an outbreak is easily created. On the other hand, students with LTBI are more likely to progress to active TB compared with other groups due to the heavy study burden, immature immune system, and reduced protective efficacy of the BCG vaccine (Williams et al., 2016).

As close contacts are one of the at-risk populations for active TB (Hu et al., 2012), it is critical to identify LTBI accurately and provide preventive treatment for them. Currently, there is no gold standard method for identifying LTBI worldwide. Either a TST or IGRA can be used to test for LTBI (World Health Organization, 2018). TST is widely used in China, but it can lead to a false-positive reaction in those vaccinated with BCG (World Health Organization, 2018). Under the National Immunization Program in the 1960s, BCG covered almost the whole country of newborns in China. We assessed the prevalence of LTBI by QFT in this study.

The prevalence of LTBI among all kinds of contacts was 51.5% (95% CI 47.1–55.8) in low-middle income countries and 28.1 (95% CI 24.2–32.4) in high-income countries (Fox et al., 2013). In this study, LTBI prevalence was 4.8% in close contacts closing to 6.2% in 17-18-year-old schoolchildren by HU et al. (Hu et al., 2013), which was much lower than reported in other studies even compared to the prevalence of local-born contacts in high-income countries (17.0%,95% CI 11.8-24.0) (Fox et al., 2013). One possible reason was the higher specificity of QFT among that BCG vaccinated (Nicol et al., 2009; Kariminia et al., 2009). In addition, a developed school TB prevention and control system in Shanghai might make a difference.

A national TB registration system is initialed in China that once a TB patient is diagnosed in a TB specialized hospital, detailed information about the patient must be registered in the system within 24 hours. Registration in the system for patients 3-18 years old, includes school information and details such as school name and address, class, ID, and phone number. A message would be sent to the local CDC. CDC staff must confirm the information received within 24 hours and start an investigation within 3 days in Shanghai, cutting off the possible further transmission in time.

Including our own, studies in Shanghai showed that LTBI was associated with exposure to infectious TB patients, no BCG vaccination, and tobacco smoke (Hu et al., 2013), The risk of contact becoming infected relates to the infectiousness of the TB patient, the duration and proximity of the contact, and susceptibility of the contact (Kenyon et al., 1996; Suggaravetsiri et al., 2003; Fok et al., 2008; Yim and Selvaraj, 2010). Bacteriologically confirmed patients were infectious, it can lead to secondary cases of active TB and LTBI by contacting with them (Velleca et al., 2021). We found the LTBI rate was higher in those in contact with bacteriologically confirmed patients. It is important to detect and cure infectious TB patients timely (World Health Organization, 2021). Diagnosis time has been shortened by using molecular biological tests. Infectious patients have to leave school and stop studying after diagnosis which helps to lessen the transmission risk. While, before the diagnosis of TB, ones getting a cough, fever, or other symptoms should have the awareness of avoiding taking part in group activities in confined spaces, and visit doctors the first time. We can build daily infection control rules for enhancing the ability of infectious diseases control and prevention. Poor lighting and ventilation conditions may increase the risk of infection. Either opening windows or installing ventilation equipment can help, and regular disinfection is equally important.

BCG vaccination can protect against both TB disease and TB infection (Soysal et al., 2005; Hu et al., 2013). The quality and quantity of antibodies produced in response to BCG may impact the immune response and contribute to early protection against *M. tuberculosis* infection (Bright et al., 2021). We identified those without BCG scars were more likely to be infected. Although the protective efficacy of BCG decreases over time (Colditz et al., 1995; Sterne et al., 1998), it may still protect college students, we suggest that the protection of BCG vaccination can decrease the rate of LTBI, BCG vaccines can be provided for unvaccinated students especially those from high TB burden areas (Hu et al., 2013).

Tobacco smoke can alter people’s mucociliary function and decrease the ability to clear inhaled substances, helping bacteria to adhere to the epithelial cells of the airways. It can also increase alveolar permeability and weaken both humoral and cellular immunity (Murin and Bilello, 2005; Feng et al., 2011). We found that smokers were more susceptible to *M. tuberculosis* even if they had quit smoking. We recommend establishing a smoke-free campus through health promotion and doing better TB control.

Close contacts were at higher risk of active TB than the general population (Guo et al., 2019), especially those smoking and overweight (Slama et al., 2007; Lu et al., 2021). In our study, the mean annual incidence of active TB was 0.2% in close contacts, which was more than 7 times compared to the incidence of permanent residents. 5% to 10% of the individuals with LTBI will develop TB disease during their lifetime, with most cases occurring within the first 5 years after initial infection, especially within the first 2 years (Lillebaek et al., 2002; Houben and Dodd, 2016; World Health Organization, 2021).

A meta-analysis identified 18 publications reporting TB incidence among 23 paired cohorts of individuals with a positive TST reaction in whom active TB was excluded. Among the LTBI cohorts, the weighted mean adjusted incidence rate of active TB in the LTBI was 13.5 per 1000 person-years (Andrews et al., 2012). In our study, the incidence rate was 21.9 per 1000 person-years in TST (+++) cohort, which was higher than in other studies.

The incidence rate of active TB among local LTBI individuals in the United States from 2006 to 2008 was approximately 0. 84/1,000 person-years (Shea et al., 2014). A cohort study of adolescent LTBI individuals by QFT in South Africa showed that the incidence rate of active TB was 6.4 per 1000 person-years (Mahomed et al., 2011). Another cohort study in rural China showed that the incidence rate of active TB was 8.7 per 1000 person-years in the QFT (+) subjects (Gao et al., 2017). In our study, the incidence rate was 14.1 per 1000 person-years in the QFT (+) cohort, which was higher than in other studies.

The follow-up results indicated that close contacts especially those with LTBI in schools are crucial for TB control, and management of LTBI in high-risk groups is a key action for the realization of the “End TB Strategy”.

Our study has several limitations that deserve further comment. First, for the short follow-up time for the cohort, we cannot realize the long-term effects. Second, as a small number of individuals progressed to disease, we cannot analyze the possible risk factors related. Our future research will include a longer follow-up period to obtain more conclusive data.

## Conclusions

This prospective cohort study revealed the prevalence of LTBI, the possible risk factors associated at baseline and tracked the development of active TB among close contacts of TB index cases in schools. Our results suggest that school close contacts are one of the key populations for LTBI management. Establishing daily infection control rules, enhancing BCG vaccination, and smoke-free campuses will further reduce the prevalence of LTBI. Preventive treatment for LTBI is an important strategy for the reduction of the incidence of TB. In addition, enhancing TB health promotion, and paying attention to those from high TB burden areas may also be helpful to reduce the incidence.

## Data availability statement

The original contributions presented in the study are included in the article/supplementary material. Further inquiries can be directed to the corresponding author.

## Ethics statement

The studies involving human participants were reviewed and approved by Ethical Review Committee at Shanghai Municipal Center for Disease Control and Prevention. The patients/participants provided their written informed consent to participate in this study.

## Author contributions

XX, PL, LL, and LT participated in the study design, XX, YJ, JL, YZ, ZW, and LR participated in the data collecting and statistical analysis, XX wrote the first draft of the manuscript. JC, TZ, ZY, and QP reviewed and revised the first draft. XS conceived, designed, and managed the study. All authors contributed to the article and approved the submitted version.

## Funding

This study was supported by the Chinese National Science and Technology Major Project (grant no. 2018ZX10715012), Shanghai Municipal Health Commission youth Project (grant no. 20214Y0480), and Shanghai Municipality Science and Technology Commission Project (grant no. 20Z11900502).

## Acknowledgments

We would like to thank all the staff in district CDCs for collecting data.

## Conflict of interest

The authors declare that the research was conducted in the absence of any commercial or financial relationships that could be construed as a potential conflict of interest.

## Publisher’s note

All claims expressed in this article are solely those of the authors and do not necessarily represent those of their affiliated organizations, or those of the publisher, the editors and the reviewers. Any product that may be evaluated in this article, or claim that may be made by its manufacturer, is not guaranteed or endorsed by the publisher.
